# Universality of universal health coverage: A scoping review

**DOI:** 10.1371/journal.pone.0269507

**Published:** 2022-08-22

**Authors:** Aklilu Endalamaw, Charles F. Gilks, Fentie Ambaw, Yibeltal Assefa

**Affiliations:** 1 School of Public Health, The University of Queensland, Brisbane, Australia; 2 College of Medicine and Health Sciences, Bahir Dar University, Bahir Dar, Ethiopia; 3 School of Public Health, College of Medicine and Health Sciences, Bahir Dar University, Bahir Dar, Ethiopia; Chung Shan Medical University, TAIWAN

## Abstract

**Background:**

The progress of Universal health coverage (UHC) is measured using tracer indicators of key interventions, which have been implemented in healthcare system. UHC is about population, comprehensive health services and financial coverage for equitable quality services and health outcome. There is dearth of evidence about the extent of the universality of UHC in terms of types of health services, its integrated definition (dimensions) and tracer indicators utilized in the measurement of UHC. Therefore, we mapped the existing literature to assess universality of UHC and summarize the challenges towards UHC.

**Methods:**

The checklist Preferred Reporting Items for Systematic reviews and Meta-analysis extension for Scoping Reviews was used. A systematic search was carried out in the Web of Science and PubMed databases. Hand searches were also conducted to find articles from Google Scholar, the World Bank Library, the World Health Organization Library, the United Nations Digital Library Collections, and Google. Article search date was between 20 October 2021 and 12 November 2021 and the most recent update was done on 03 March 2022. Articles on UHC coverage, financial risk protection, quality of care, and inequity were included. The Population, Concept, and Context framework was used to determine the eligibility of research questions. A stepwise approach was used to identify and select relevant studies, conduct data charting, collation and summarization, as well as report results. Simple descriptive statistics and narrative synthesis were used to present the findings.

**Results:**

Forty-seven papers were included in the final review. One-fourth of the articles (25.5%) were from the African region and 29.8% were from lower-middle-income countries. More than half of the articles (54.1%) followed a quantitative research approach. Of included articles, coverage was assessed by 53.2% of articles; financial risk protection by 27.7%, inequity by 25.5% and quality by 6.4% of the articles as the main research objectives or mentioned in result section. Most (42.5%) of articles investigated health promotion and 2.1% palliation and rehabilitation services. Policy and healthcare level and cross-cutting barriers of UHC were identified. Financing, leadership/governance, inequity, weak regulation and supervision mechanism, and poverty were most repeated policy level barriers. Poor quality health services and inadequate health workforce were the common barriers from health sector challenges. Lack of common understanding on UHC was frequently mentioned as a cross-cutting barrier.

**Conclusions:**

The review showed that majority of the articles were from the African region. Methodologically, quantitative research design was more frequently used to investigate UHC. Palliation and rehabilitation health care services need attention in the monitoring and evaluation of UHC progress. It is also noteworthy to focus on quality and inequity of health services. The study implies that urgent action on the identified policy, health system and cross-cutting barriers is required to achieve UHC.

## Introduction

Universal health coverage (UHC) is a multi-dimensional concept that includes population coverage, services coverage and financial protection as its building blocks, as well as equity and quality in its integrated definition [1]. Health policy and decision makers believe UHC as a foundation to improve population’s health, facilitate economic progress, and achieve social justice [2, 3]. It is also essential to minimize disparities, promote effective and comprehensive health governance, and build resilient health systems [4].

The United Nation’s (UN) post-2015 goals described UHC as the predominant approach to realize the 2030’s sustainable health goals [5]. It is also taken as an urgent priority in 2020 UHC high-level meeting to address global health crises, through delivering affordable essential quality healthcare services, including the pandemic COVID-19 [6]. The UN General Assembly further declared, at its 73^rd^ session, that global institutions and countries make healthcare accessible to one billion more people by 2023 [7] and 80 percent of the population by 2030 with no catastrophic health expenditures [5].

WHO and the WB established core tracers of health service coverage to monitor UHC [8]. These tracers are categorized under the main theme reproductive, maternal, neonatal, and child health (RMNC), infectious diseases (IDs), non-communicable diseases (NCDs), and service capacity and access (SCA). Another dimension of UHC in SDG 3.8.2 is financial risk protection, which is typically measured by catastrophic health expenditure (CHE) and impoverishment due to healthcare costs [8].

While no prior studies have been conducted to identify and map the available evidence on UHC, other related studies such as “a synthesis of conceptual literature and global debates” [1] and a scoping review of “implementation research approaches of UHC” [9] are available. In addition to these literature, another study assessed the hegemonic nature of UHC in health policy described historical background of how UHC emerged, and frequency of UHC mentioned in all fields of articles available in PubMed database [10]. None of those previous studies addressed the universality of UHC in terms of its building blocks and service types and summarized the findings from each study included in the review.

A scoping review of the studies on UHC and its dimensions is crucial to map and characterize the existing studies towards UHC. This will help to identify key concepts, gaps in the research, and types and sources of evidence to inform practice, policymaking, and research [11]. The goals of this scoping review towards universality of UHC were, first, to determine the distribution of articles across WHO and WB regions, health service types, and dimensions including major components and tracer indicators, and second, to synthesize barriers of UHC. This review provides insight that is useful in setting strategies, evaluating health service performance, and advancing knowledge on priority research questions for future studies.

## Methods

### Identifying a research question

The protocol of this scoping review is available elsewhere https://doi.org/10.21203/rs.3.rs-1082468/v1. The overall activities adhered to the Arksey and O’Malley’s (2005) scoping review framework [12], which was expanded with a methodological enhancement for scoping review projects [13], and the Joanna Briggs Institute framework [14]. The review followed five steps: (1) identifying research questions, (2) identifying relevant studies, (3) study selection, (4) data charting, and (5) collation, summarization, and reporting of results. The checklist Preferred Reporting Items for Systematic reviews and Meta-analysis extension for Scoping Reviews were used (S1 Checklist) [15].

The research questions were developed by AE in collaboration with YA. The Population, Concept, and Context framework was used to determine the eligibility of research questions [16]. According to the framework, the population represented study participants to whom findings infer which includes people at any age or other important characteristics of study participants. Not all UHC expected to have population component, which is non-applicable in some research. The concept was overall UHC or financial risk protection, equity, quality, and coverage. Context includes the study settings or countries and, in this review, the global context.

### Identifying relevant studies

Web of Science, PubMed and Google Scholar were used to find literature in the field. Hand search was also used to find articles from WB Library, WHO Library, UN Digital Library Collections, and Google. Using the relevant keywords and/or phrases, a comprehensive search strategy was established. Universal, health, "health care", healthcare, "health service”, quality, access, coverage, equity, disparity, inequity, equality, inequality, expenditure, and cost were search words and/or phrases. “AND” or “OR” Boolean operators were used to broaden and narrow the specific search results. Search strings were formed in accordance with the need for databases (S1 Table). Article search date was between 20 October 2021 and 12 November 2021, with the most recent update on 03 March 2022. The articles were imported into EndNote desktop version x7, which was used to perform an automatic duplication check. Manual duplication removal was also performed. The database search strategies are shown in the (S1 Table).

### Study selection

In consultation with YA, AE developed and tested study selection forms (inclusion and exclusion criteria) using a random sample from collected references, which were found using search strategy. A second meeting was held to approve the study screening form and process. Then, inclusion and exclusion criteria were applied during the article screening process for all articles. Studies conducted using the English language were included. Articles on overall UHC (UHC effective service coverage and FRP), UHC effective service coverage, UHC without specification with service coverage and FRP, and which reported coverage, quality, inequity, FRP in the outcome of the study or explored UHC research objectives were included. Types of study design included were quantitative, qualitative, mixed-research, and review types. The search was narrowed to include only literature published since 2015 to find studies which addressed the SDG target 3.8 and proceeding years. Non-English language literature, abstracts only, comments or letters to the editor, erratum, corrections, and brief communications were all excluded.

Articles’ titles, abstracts, and full texts were reviewed in stages. After duplicates were removed using EndNote desktop x7 software and manual duplication removal, titles were screened. After that, abstracts were used to screen the literature. Those who passed abstract review were eligible for full-text review. Full-text articles were also screened for data charting purposes. For articles with only an abstract, contact was made with the study’s corresponding authors.

### Data charting

A piloted and refined data extraction tool was initially developed to chart the results of the review from full-text literature. Data was examined, charted, and sorted according to key issues and themes. Author(s), publication year, WHO geographic category, WB group, study approach, studied domain or topic, UHC themes, and health care service types were all extracted.

### Collation, summarization, and report of results

Based on years of publication, studied dimensions (interrelated objectives), WHO region, WB group, study approach, and health care service types, available articles were compiled and summarized with frequency and percentage.

A simple descriptive analysis was performed, and the results were presented in the form of tables and figure. The data reporting scheme was adjusted as needed based on the findings.

## Results

### Search results

**Fig 1:**
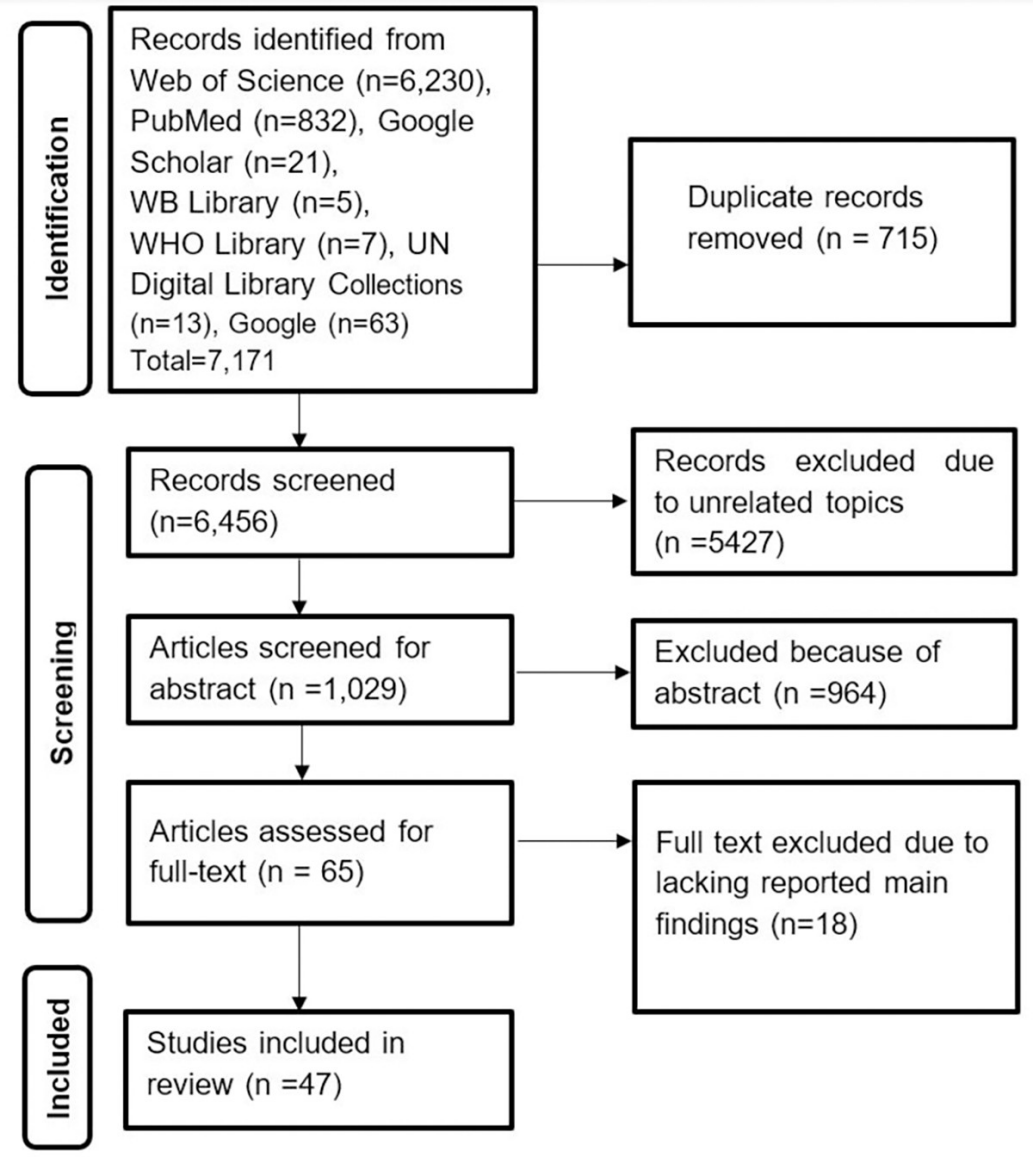
PRISMA-ScR flow diagram for articles selection process.

PubMed (n = 6,230) and Web of Science (n = 832) databases were searched. Google Scholar (n = 21), WB Library (n = 5), WHO Library (n = 7), UN Digital Library Collections (n = 13), and Google (n = 63) were also manually searched. A total of 7,171 records were discovered. Following title and abstract screening, 65 articles were chosen for full-text review. Finally, 47 articles were selected for scoping review (Fig 1).

### Articles characteristics

Almost one-fourth of articles were from WHO Africa region and another 25.5% were across two or more WHO regions. According to income category, 42.5% were from lower-middle-income countries followed by 29.8% across two or more WB economy groups. More than half of the articles (54.1%) followed a quantitative research approach (Table 1). The countries where each article conducted are available in S2 Table.

**Table 1:** Articles distributions by World Health Organization region and World Bank category, study approach, and year (n = 47).

Variables	Frequency	Percentage %)
**World Health Organization regions**		
Across WHO regions	12	25.5
Africa	12	25.5
Americas	5	10.6
Eastern Mediterranean	4	8.5
South-East Asia	9	19.1
Western-Pacific	5	10.6
**World Bank Categories**		
Across World Bank groups	14	29.8
Lower-Middle-income	20	42.6
Low-income	3	6.4
Upper-middle-income	10	21.3
**Study approach**		
Quantitative	25	53.2
Qualitative	13	27.6
Review	2	4.3
Mixed approach	7	14.9
**Publication Year**		
2015	1	2.1
2016	7	14.9
2017	4	8.5
2018	7	14.9
2019	12	25.6
2020	9	19.1
2021	7	14.9

FRP-Financial risk protection; HIV/AIDS- Human immunodeficiency virus/acquired immunodeficiency syndrome; UHC-universal health coverage; WHO-World Health Organization

### Health service types

Twenty articles [17–36] are categorized under health promotion. These articles were focused on pathways and efforts, program evaluation and change, opportunities and challenges, barriers/factors/enablers, community-based health planning and service initiative, perceived effect of health reform on UHC, health-seeking behaviour and knowledge, health security and health promotion activities, the impact of insurance on coverage, and SCA dimensions of UHC. Health promotion encompasses funding and infrastructure, health literacy, the development of healthy public policies, the creation of supportive environments, and the strengthening of community actions and skills, as well as any activities that assist governments, communities, and individuals in dealing with and addressing health challenges.

Six articles discussed treatment aspects of health services, which were access to care for illness, access to treatment for rheumatic heart diseases, neglected tropical diseases (NTDs), mental disorders and hypertension [37–42].

According to GBD-2019, WHO and WB tracers, FP and/or SCA components for promotion, immunization for prevention and other diseases in RMNC, IDs, NCDs for treatment aspects, nineteen articles were a combination of promotion, prevention, and treatment aspects [43–61].

One study looked at both the promotion and treatment of health services [62].

One study done on the quality of health care for disabled people [63] was classified as a palliative and rehabilitative care service type despite it did not adhere to palliative care assessment guidelines.

### Components and dimensions of UHC

The main four components of UHC are RMNC, IDs, NCDs and SCA. Of included articles, RMNC was reported by 19 articles, 17 assessed NCDs were reported by 17 articles, CDs was assessed by 13 articles, and SCA was assessed by 9 articles. Regarding dimensions, coverage was assessed by 25 of articles; FRP by 13 articles, inequity by 10 articles and quality by 3 articles (S2 Table).

### Tracer indicators for summary measure of UHC

Of 25 quantitative articles, 19 articles used various tracer indicators to assess UHC quantitatively; the remaining six quantitative articles assessed each empirical analysis of the potential impact of importing health services, access and financial protection of emergency cares, perceived availability and quality of care, the performance of district health systems, crude coverage and financial protection, health-seeking behaviour and OOP health expenditures, and the performance of health system.

Accessibility and affordability in China [64], as well as curative care and quality of care components in India [48] were developed as new tracers.

Ten tracers were used in RMNC component of UHC. Five tracers in IDs, seven tracers in SCA and 18 tracers were used NCDs component of UHC. Three tracers were used for FRP estimation. The iteration of tracers under four components of UHC effective service coverage and FRP is shown in Table 2.

**Table 2:** Tracer indicators used by studies to estimate UHC.

Components Tracers	Number of articles	Components Tracers	Number of articles
**Reproductive, Maternal, Neonatal and Child Health**		**Non communicable disease**	
Immunizations [43, 46–52, 54–61]	14	Fasting blood glucose/ diabetes treatment [43, 44, 54–60]	9
ANC and delivery [43, 44, 46–52, 55–57, 59–61]	15	Non-use of tobacco [43, 49, 52, 55–60]	9
Family planning [43, 46, 48– 50, 52, 54–60]	13	Non-raised blood pressure [43, 44, 55–60],	8
Child care seeking for pneumonia [46, 47, 49–52, 54–57, 59–61]	13	Cervical cancer screening/treatment [44, 51, 54, 55, 59–61]	7
Under-5 diarrhoea treatment [44, 47–52, 54, 61]	9	Breast cancer screening/ treatment [44, 51, 54, 61]	4
Skilled birth attendance [43, 44, 46, 47, 49–52, 61]	9	Uterine cancer treatment [54]	1
Perinatal care for newborn babies and mothers [44, 48, 49, 54]	4	Colon and rectum cancer treatment [54]	1
Exclusive breastfeeding [46, 49, 52]	3	Non-overweight [49]	1
Iron and folic Acid (≥100) [48]	1	Acute lymphoid leukaemia treatment [54]	1
Tetanus toxoid [48]	1	Asthma treatment [54]	1
**Infectious diseases**		Epilepsy treatment [54]	1
Water and adequate [43, 49, 50, 52, 55–60]	9	Appendicitis treatment [54],	1
Tuberculosis effective treatment [43, 54–60]	8	Paralytic ileus and intestinal obstruction treatment [54]	1
Antiretroviral Therapy [43, 54–60]	8	Ischemic heart disease treatment [54]	1
Insecticide-treated bed nets [46, 52, 55, 59, 60]	5	Stroke treatment [54]	1
Tuberculosis case detection [43]	1	Chronic kidney disease treatment [54]	1
**Service Capacity and Access**		Chronic obstructive pulmonary disease treatment [54]	1
Health worker density [55–60, 64]	7	Provision of treatment or advice on physical activity or diet [37]	1
Hospital bed density [55–60]	6	**Financial Risk Protection**	
Health security [55–60]	6	Catastrophic healthcare spending [37, 43, 47–52, 58, 61, 64]	11
Access to essential medicine [55, 59, 60, 64]	4	Impoverishment by out of pocket healthcare spending [43, 49, 50, 52, 58]	5
Inpatient admission [47, 61]	2	Poverty gap due to healthcare spending [58]	1
Utilization of outpatient services [37, 64]	2		
health status after the last outpatient visit [37]	1		

### Barriers/challenges of UHC

Policy, health sector and cross-cutting barriers of UHC were identified. Financing, leadership/governance, inequity, regulation and supervision mechanism, and poverty were most repeated policy level barriers. Poor quality health services and inadequate health workforce were the common barriers from health sector challenges. Lack of common understanding on UHC was frequently mentioned cross-cutting barriers (Table 3).

**Table 3:** Policy, health sector and cross-cutting barriers of UHC.

Policy level barriers	Health sector level barriers	Cross-cutting barriers
Financing system [18, 21, 22, 24, 27–29]	Human resources shortage [28]	Lack of empowerment and information [28]
Governance and leadership [17, 18, 24, 28, 31, 32]	Deficient training [21, 28]	Disease pattern [29]
Regulation and supervision mechanism [28, 29, 31]	Low motivation [28]	People perception that health care should be free of cost [28]
Poverty [22, 29, 35]	Staff retention [28]	Health profile disparities between districts [32]
Inequity in income or service coverage [22, 24, 27, 32, 35]	Skill-mix imbalance [28]	Unhealth life style [42]
Lack of attention to marginalized population [28]	Service delivery [18]	Behaviour [29, 42]
Insurance-related problems [22, 27]	Health care quality problem [21, 28, 32]	Lack of common Understanding on UHC [17, 28, 33],
Accreditation of facilities [21]	Lack of guideline [28]	Global movements [18]
Narrowness of the benefit package [21]	Political interference [28]	Population health status [18]
Inadequate multi-sector collaborations [24]	Ineffective monitoring and supervision [22, 28]	Coverage [18]
Donor driven vertical programs [24]	Professional recruitment mechanisms [28]	Social determinants of health [32]
Unpreparedness [27]	Inadequate number of health workers [21, 24, 31],	Emerging of non-communicable diseases [35]
Social infrastructure and social sustainability [18]	Absence of diagnosis of the priority demands or conflict in setting priority [31, 33]	Technology and equipment [31, 42]
Failures in the expansion/shortage of services [31, 42]	Inadequate health system to early diagnose [42]	
Poor infrastructure [21, 31, 42]	Delayed reimbursement using prepaid health insurance [42]	

## Discussion

The purpose of this scoping review was to map existing research, and the most researched UHC dimensions, components and summarized main findings. Many articles were found in the African region and in countries with middle-income (lower and upper). Many of the studies followed a quantitative research approach. Palliative and rehabilitative health care types did not be well address in UHC research. The service coverage and financial protection dimensions were most frequently studied, followed by inequity and quality of health care services.

The current evidence found a greater number of articles than a scoping review of African implementation research of UHC [65]. This is because the former was conducted on a single continent and concentrated on UHC research approaches. Another bibliometric analysis, on the other hand, discovered a greater number of available evidence than the current scoping review [66]. Because it includes all available evidence as terminology, title, phrases, or words in policy documents, commentaries, editorials, and all frequency counts found in databases by the first search without the conditions of pre-established exclusion criteria. Aside from that, the bibliometric analysis included articles dating back to 1990. UHC is a global agenda that has improved the health of the global population through political support, funding, and active national and international collaborations [67, 68]. The number of research output is likely increasing over time though current evidence shows that comparable numbers of articles are available in each year. An earlier bibliometric analysis discovered an increasing trend of UHC research outputs [66].

Many of the studies in this review used a quantitative approach. A prior scoping review conducted in Africa discovered that qualitative and mixed-methods studies were the commonest method to investigate UHC [10]. The former study did not consider financial protection research, UHC effective service and crude coverage, service capacity and access. UHC is intended to be quantified numerically as a summary index to track the progress of health care performance. Given the nature of UHC, fewer articles used qualitative research design to investigate its challenges, opportunities, and success of UHC. Various health systems and policies in low, middle, and high-income countries may present different barriers and facilitators to achieve UHC [69–71]. The current review has also identified policy, health system and cross-cutting barriers of UHC that were frequently explored by qualitative research.

Many number of countries are available in the European region and the high-income category [72, 73]. In contrast, a substantial amount of UHC research was produced in middle-income countries, most were from African region. Trend analysis in health policy and systems research conducted on the overall research progress discovered that an increasing trend of publications in low-and middle-income countries between 2003 and 2009 [74]. This could be attributed to the nature of the health problems and the health policy in place regarding health research. Furthermore, health research budgets and clinical trial infrastructures may determine health research activities in each continent. Health budget might not always true in its effect of high research publication. For instance, evidence from a review finding indicated that nations with significant donor investment in health research may not necessarily produce a large number of research [75]. Articles available across WHO regions were comparable to frequency of articles in WHO African region. This might be due to UHC is a global strategy in monitoring the global process towards universal access to health care. The availability of UHC monitoring framework helps to conduct to conduct research at the multicounty level.

In 2019, the burden of NCDs was 63.8 percent worldwide, followed by IDs, RMNC, and nutritional disease (26.4 percent) [76]. In the summary measure of the UHC index, RMNC was the most frequently studied component followed by NCDs. This could be because many of the articles in the current review came from Africa and lower-and middle-income countries. In these countries, maternal and child morbidity and mortality were extremely high [77], making RMNC more likely to be investigated in UHC context. Similarly, a scoping review study on maternal, neonatal, and child health realized a high rate of publication in the most recent period [78].

This review provides an answer to the question of how much UHC is universal and how much UHC is covered in the current health systems and policy research. UHC tracer indicators are focused on health promotion, disease prevention, treatment, palliative, and rehabilitative health care services at the individual and population level. Promotion aspects of health services were more frequently investigated in the current review. This could be because those articles non-specific to either component of UHC were classified as health promotion. A single study was conducted on disabled population, close to palliative and rehabilitative health care types. Palliative care focuses on the physical, social, psychological, spiritual, and other issues confronting adults and children living with and dying from life-limiting conditions, as well as their families [79]. Assessment of pain and symptom management, functional status, psychosocial care, caregiver assessment, and quality of life are all part of a palliative care measurement and evaluation domains [80]. The Worldwide Hospice Palliative Care Alliance recommended research to improve palliative care coverage [81] in order to ensure equitable health care access for more than 40 million people who require palliative care each year worldwide [82]. However, UHC effective service coverage measurement indicators are appropriate only for assessing the promotion, prevention and treatment aspects of health care, even though all health care services are theoretically expected to be covered [54].

In terms of dimensions, coverage was more commonly studied. The framework for monitoring and tracking was initially established for effective service coverage and FRP. UHC’s service coverage is a collection of many individual disease indicators used to assess the performance of the health care system. Therefore, it is not surprising that many articles have been written about the coverage dimension. Aside from the usual trend of calculating the service coverage summary index, a few articles estimated UHC by combining effective service coverage and FRP indicators. In the current review, a few studies assessed the quality of care as a dimension of UHC; a single study developed a distinct quality of care measurement that integrated into the UHC matrix. Effective service coverage is predicated on the assumption that those in need receive high-quality health care services. Effective services coverage and quality are theoretically integrated. However, having a high UHC index value does not imply that high-quality care is provided for each specific disease. For example, in countries with high UHC index value [54], quality medical care services were found to be inadequate for patients with chronic diseases [83]. Quality of health care can be assessed using structure, process, and outcome indicators in the healthcare system [84].Therefore, generally, measuring the quality of care for specific disease is helpful for stakeholders, clinicians, and health policymakers working on specific health problems [85].

The UHC summary index is also useful in comparing the national and subnational progress of health system performance between countries and within a specific country. One of UHC’s primary functions is to promote health equity [3], and equity has been identified as a measurable component of UHC [86]. It is linked to social determinants that should be monitored over time, across or within different settings and populations [87]. Inequity in UHC service coverage studies was reported broadly. None of the UHC articles examined health disparities based on age, gender, race or ethnicity, residence, education level, or socioeconomic status. Moreover, range, absolute or relative difference, concentration index, and Gini coefficient were not used as equity measurement techniques in the included articles.

As implication to policy/program manager and researcher, more research is needed in settings where UHC has not been thoroughly investigated qualitatively. Future research better focus on the quality and equity dimension of UHC health care services. Given that the distinct nature of UHC tracers may limit UHC’s articles on health promotion, prevention, and treatment aspects, palliative and rehabilitative care services require attention in the future research environment. For specific health problems, additional review may be required to identify research gaps in specific tracer.

## Strength and limitation

This is the first scoping review of UHC, and it is accompanied by the most recent articles. Our review identified UHC literature in each category of health service type.

In terms of limitations, this review included only articles conducted in English; articles conducted in other languages may have been missed, and geographical representation of UHC articles may have been over or underestimated for regions. When considering UHC dimensions, they may have a different level of research articles discovered if another mapping review is done for specific disease types.

## Conclusions

Most articles were from Africa, across WHO regions and middle-income countries. Quantitative research approach has been frequently used. Equity and quality of services have got little attention in UHC research. Palliation and rehabilitation health services have also got little attention in the UHC research. Tracer indicators other than WHO and WB were developed and utilized in different countries. Policy, health sector and cross-cutting barriers of UHC were identified. Financing, leadership/governance, inequity, regulation and supervision mechanism, and poverty were most repeated policy level barriers. Poor quality health services and inadequate health workforce were the common challenges of the health sector towards UHC. Lack of common understanding on UHC was frequently mentioned as cross-cutting barrier.

## Supporting information

S1 Checklist Items followed in conducting this review. (DOCX)

S1 Table Search strategy. (DOCX)

S2 Table Articles’ country and main findings. (DOCX)

## Abbreviations

ANC: Antenatal Care
AR: Antiretroviral Therapy
CHI: Catastrophic Health Expenditure
FP: Family Planning
GBD2019: Global Burden Disease 2019
HIV/AIDS: Human Immunodeficiency Virus/Acquired Immunodeficiency Syndrome
IDs: Infectious diseases
NCDs: Non-communicable diseases
RMNC: Reproductive, Maternal, Neonatal and Child
SCA: Service Capacity and Access
SDGs: Sustainable Development Goals
UN: United Nations
UHC: Universal Health Coverage
WB: World Bank
WHO: World Health Organization
