# Correction: Universality of universal health coverage: A scoping review

**DOI:** 10.1371/journal.pone.0304023

**Published:** 2024-05-16

**Authors:** Aklilu Endalamaw, Charles F. Gilks, Fentie Ambaw, Yibeltal Assefa

The affiliation for the second and fourth author is incorrect. Charles F. Gilks and Yibeltal Assefa are not affiliated with #2 but with #1: School of Public Health, The University of Queensland, Brisbane, Australia.
