# Supplementary material for: Universality of universal health coverage: A scoping review
Source: PLoS One. 2022 Aug 22;17(8):e0269507. doi: 10.1371/journal.pone.0269507 (PMC9394787; doi:10.1371/journal.pone.0269507)
Supplement: S1 Table — (DOCX) [file pone.0269507.s002.docx]

| Database | Search strategy | Result | Filters applied: |
| --- | --- | --- | --- |
| PubMed | (Universal[Title/Abstract] AND (health[Title/Abstract] OR "health care"[Title/Abstract] OR healthcare[Title/Abstract] OR "health service*"[Title/Abstract])) AND (access[Title/Abstract] OR coverage[Title/Abstract] OR Equity[Title/Abstract] OR disparity[Title/Abstract] OR inequity[Title/Abstract] OR equality[Title/Abstract] OR inequality[Title/Abstract] OR quality[Title/Abstract] OR expenditure [Title/Abstract] OR cost [Title/Abstract]) Filters applied: English, Humans, from 2015/1/1 - 2022/3/3. | 6,230 | From 2015/1/1 - 2022/3/3 |
| Web of Science | (TI=(“Universal” ) AND TI=(“health” OR "health care" OR “healthcare” OR "health service") AND TI=(access OR coverage OR equity OR disparity OR inequity OR equality OR inequality OR quality OR expenditure OR cost)) AND (PY==("2021" OR "2020" OR "2019" OR "2018" OR "2016" OR "2015" OR "2017")) AND English (Languages) Not Document type: Letters or Meeting Abstracts or Editorial Materials or Corrections or Book chapters or Books Or Book reviews or Biographical-Items | 832 | From 2015/1/1 - 2022/3/3 |
