# Supplementary material for: Universality of universal health coverage: A scoping review
Source: PLoS One. 2022 Aug 22;17(8):e0269507. doi: 10.1371/journal.pone.0269507 (PMC9394787; doi:10.1371/journal.pone.0269507)
Supplement: S2 Table — (DOCX) [file pone.0269507.s003.docx]

| Author | Country | UHC components | Dimensions | Objective | Main findings |
| --- | --- | --- | --- | --- | --- |
| Assan A et al 2019 | Ghana | Not specific | Non specific | Explore the challenges of UHC | - Inadequate understanding of concept, political and policy change |
| Barasa E et al 2018 | Kenya | RMNC, HIV, FRP | Coverage | Develop a summary measure of UHC and track the country’s progress | - UHC weighted using Service (SC) and FRP - Increased service SC and reduced percentage of FRP - Increased weighted summary measure of UHC |
| Derakhshani N et al 2021 | Iran | Not specific | Non specific | Identify the influential factors of UHC | - Financing, payment system, organization, regulation and supervision , behavior, and Others |
| Eckhardt M et al 2019 | Ecuador | Not specific | Non specific | Explore the perceived effects of health reform on UHC | - The effects of the reform were perceived as positive and the reform has improved rural primary health care services |
| Hogan DR et al 2017 | 183 countries | RMNC, CD, NCD, SCA | Coverage and equity | Presents methods and baseline results for UHC index | - Service coverage varies widely between countries, highest in East Asia and Northern America and Europe, lowest in Sub-Saharan Africa followed by Southern Asia. - Coverage of essential services has increased. |
| Hanlon C et al 2019 | Ethiopia | NCDs | Financial protection | Propose strategies to achieve UHC | - Expansion of public health insurance, leveraging resources from high-priority SDG-related programmes and implementing task-shared care |
| Koduah A et al 2021 | Ghana | NCDs | Non specific | Explore challenges of UHC | - Population, patient, and health system-related factors |
| Leslie HH et al 2019 | Mexico | RMNC and NCDs | Coverage and equity | Estimate effective coverage and its regional inequalities and to identify the challenges in generating estimates of health system performance | - Effective coverage level varied between disease; low for childhood diarrhoea to a high for newborn care. - Inequality in effective coverage existed between states. - Effective SC of these six conditions was moderate (49%) |
| Liu X et al 2021 | China | UHC new version | Coverage and financial protection | Develop a Chinese version of UHC and progress | - Indices of accessibility and affordability developed as new version - Absolute accessibility had the most significant improvement, and the index of relative accessibility decreased. - The index of absolute affordability fluctuated and relative affordability increased. |
| Mao W et al 2020 | China & Veitnam | Not specific | Coverage and equity | Different pathways to achieve UHC | - China by strong political and financial subsidies, UHC varied across regions and schemes. - Vietnam by prioritizing the poor and the near-poor, approaching more equitable manner |
| GBD 2019 Universal Health Coverage Collaborators 2020 | 204 countries | RMNC, CDs and NCDs | Coverage and equity | Assess UHC effective coverage | - Globally, performance on the UHC effective coverage index improved, yet disparity between countries, higher in Japan and Iceland to lower Somalia and the Central African Republic. |
| Okech TC et al 2016 | Kenya | Not specific | Non specific | Review initiatives of UHC | - Initiatives: commitment towards UHC - Challenges: minimal solidarity in health care financing, cases of dysfunctionalilty of health care system, minimal opportunities for continuous medical training, quality concerns in terms of stock-outs of drugs and other medical supplies, dilapidated health infrastructure and inadequate number of health workers. |
| Ranabhat CL et al 2020 | 118 countries | RMNC, CDs, NCDs and SCA | Coverage | Explore basic structural factors of UHC | - Government health expenditure, governance index, stability index, the SDI index, and GNI per capita have positive impact on UHC - Populations below poverty line negatively affect UHC index |
| Ranabhat CL et al 2019 | Nepal | RMNC, CDs and NCDs | Non-specific | Explore challenges and opportunities of UHC | - Opportunities: institutional provision, global support, progress on the health insurance act, decentralization of health service, increasing service coverage, political commitment, a sense of national priority and international support - Challenges: existing volunteer types of health insurance, misleading role of trade unions and high proportion of population |
| Reid M et al 2020 | 183 countries | RMNC, CDs, NCDs and SCA | Coverage | Determine useful tracers of UHC | - Health work force density ranked first, provision of basic sanitation and access to clean water ranked second, and provision of basic antenatal services ranked third. - World Bank income criteria, health work force density ranked first in Lower Middle Income countries and third in Upper Middle Income-Countries |
| Reid M et al 2019 | 183 countries | RMNC, CDs, NCDs and SCA | Coverage | Evaluate the extent to which TB incidence and mortality explain the variability in UHC service coverage scores | - TB incidence rate and TB mortality rate were negatively correlated, with UHC SCI score - TB incidence rates explained 45% and TB mortality rate explained 55% of variability - TB coverage ranked ninth overall, third in low income countries and less important in high income countries |
| Scammell K et al 2016 | 8 countries | RMNC | Quality and financial protection | A landscape analysis of UHC | - Government expenditure does not align with political aspirations. - Quality of care is often low and the poorest fare worst |
| Shan L et al 2017 | China | Not specific | Non specific | Determine the performance of the health insurance system and identify challenges of UHC | - About 45% of the respondents believed that there is a long way achieve UHC - Challenges: financial protection, healthcare inequity, poor portability and ineffective supervision and administration of funds |
| Singh T et al 2016 | India | Not specific | Financial protection | Ascertain health seeking behavior and out-of-pocket health expenditures | - Nearly 14.2% of the household expenditure - Out-of-pocket expenditure was mostly due to medicines followed by diagnostic and laboratory tests. - Catastrophic expenditures higher in the poorest |
| Tadesse AW et al 2021 | Ethiopia | Not specific | Non specific | Document Ethiopia’s efforts to align UHC, health security (HS) and health promotion and their implementation within its health system | - Challenges: fragmentation of health system primarily manifested as inequities in access to health services, low health workforce and limited capacity to implementation guidelines. - Donor driven vertical programs, multiple modalities of health financing, and inadequate multisectoral collaborations were key features of fragmentation |
| Tao W et al 2020 | China | RMNC, CDs, NCDs and SPA | Quality and financial protection | Analyzing the overview of UHC | - Gaps in service quality and a requirement for ongoing strengthening of financial protections. - Challenges: fragmented and inequitable health delivery system, and the increasing demand for high- quality and value- based service delivery |
| Umeh CA 2018 | Ghana, Kenya, Nigeria and Tanzania | Not specific | Non specific | Review the challenges of UHC | - Challenges: large percentage of people under poverty and unable to pay premiums, large informal sector whose members are mostly uninsured, high dropout rate from insurance schemes, poorly funded primary health care system, and fragmented health insurance fund pool |
| Wu R et al 2020 | China | Not specific | Coverage and financial protection | Impacts of private health insurance on expanding coverage, increasing access to healthcare, and financial protection | - Private insurance users lesser in utilization of healthcare services - No evidence that being member of private insurance ensured financial protection |
| Agustina 2019 | Indonesia | Not specific | Non specific | Describes the innovative UHC initiative | - Meeting national health system requirements and decentralized at district level - Challenges: high stunted growth in children and high maternal and neonatal deaths |
| Ayub A et al 2018 | Pakistan | Not specific | Coverage | Analyze “Sehat Sahulat Program towards UHC | - Considers half of poor population - Covers all secondary and limited tertiary services - Services delivered through a mix of public-private providers |
| Derakhshani N et al 2020 | low- and middle-  income counties | Not specific | Non specific | Identify potential process barriers and enablers of UHC | - Factors: social infrastructure and social sustainability, financial and economic infrastructures, population health status, service delivery, coverage, stewardship/governance, and global movements |
| Fitriana EN et al 2019 | Indonesia | Not specific | Non specific | Describe the importance of “Jaminan Kesehatan Nasional Policy Socialization” towards UHC | - Perception about the programme differ between community and the government - The government said that they had conducted effective socialization - The community said that the information provided has not been effective and have no adequate information about the programme |
| Folayan MO et al 2021 | 83 countries | RMNC, CDs, NCDs and SCA | Coverage | Determine the association between UHC, health expenditure and the global prevalence of early childhood caries (ECC) | - No association between UHC service coverage index and ECC |
| França VH et al 2016 | Brazil | Not specific | Non specific | Investigate the knowledge of managers and health professionals, social workers and education professionals regarding the principal barriers of UHC | Barriers: failures in the expansion and strengthening of the services, absence of diagnosis of the priority demands, shortage of technology, equipment, and material and human resources, poor local infrastructure, and actions with low resolute power and absence of interdepartmental policies |
| Fusheini A 2016 | South Africa | Not specific | Non specific | Explore the opportunities and challenges of UHC | Opportunity: Progress on toward UHC reform, emphasis on districts and positive discrimination to narrow health inequities  Challenges: disparities across districts in relation to health profiles, health delivery performance, district management capacity, income levels, non-compliance with quality standards |
| Goepple C et al 2016 | China, Ghana, India, Mexico, the Russian  Federation and South Africa | NCDs and FRP | Coverage, equity and financial protection | Assess universal health coverage | - The weighted proportion access to basic chronic care varied between countries - Disadvantaged poor people, except in South Africa where primary health care is free to all. - Catastrophic out-of-pocket expenditure varied between countries lowest in China and highest in in Ghana, common in the poorest individuals - Health insurance increased access to care, but unable to prevent financial hardship |
| Joarder T et al 2019 | Bangladesh | Not specific | Non specific | Explores the existing health policy environment, current activities and challenges of UHC | - Policy: comprehensive set of policies for UHC - Activities: Roll out of the essential package of health services for all, expansion of access to primary health care services, and the piloting of health insurance - Barriers: rigid public financing structure dating from the colonial era, human resources, political interference, monitoring, and supervision, sociocultural disinclination, historical mistrust, and lack of empowerment |
| Nguhiu PK et al 2017 | Kenya | RMNC and CDs | Coverage and equity | Assess the extent to which the health system provides effective and equitable health services towards universal health coverage | - SC has increased, but difference between diseases - SC disparity between income status reduced except maternal health services |
| Nikoloski Z et al 2021 | Myanmar | RMNC and SPA | Coverage, equity and financial protection | Evaluation of progress toward universal health coverage | - Negative correlation between the UHC index and poverty levels. - Service accessibility pro-rich - Poor are at a greater risk of suffering financial catastrophe |
| Oraro-Lawrence T 2020 | Kenya | Not specific | Non specific | Identify similarities and differences of opinion amongst stakeholders on the challenges of UHC | - Lack of agreement on stakeholders’ interpretation of UHC, contextual values and priorities - Most interviewees prioritising an equity-based approach - Conflicting between key stakeholders in setting priority |
| Prinja S et al 2020 | India | RMNC | Coverage, equity and financial protection | Develop a composite indicator to measure UHC | - Preventive and curative services indices is presented; summary index (CUHCI) for UHC varied between regions - There was low unmet need for curative care - High incurred catastrophic health expenditures, most among the poorest |
| Rahman MS et al 2018 | Bangladesh | RMNC, CDs and NCDs | Coverage, equity and financial protection | Understand the progress of UHC by 2030 | - Part of disease’ tracers will reach 80% coverage by 2030, and others could not be achieved. - Disparity based on income will continue - Financial risk will decrease by 2030, but the wealthiest households would disproportionately face more financial catastrophe |
| Taniguchi H et al 2021 | Iraq | RMNC | Coverage, equity and financial protection | Examine inequality by 2030 and determinants of UHC | - Inequality indices will decrease by 2030 - The wide inequalities are projected to remain in DTP3, measles, full immunisations, and antenatal care in 2030. - The pro-rich inequality gap in catastrophic health expenditure will increase - Factors: mothers’ higher education and more antenatal care visits increase SC, while higher number of children and elderly population in the households increase risk of catastrophic and impoverishment |
| Trani J et al 2017 | Afghanistan | NCDs | Coverage and quality | Investigate whether a decade of international investment in the Afghan health system has brought quality health care to this group | - Formal education, higher asset level, being employed associated with improved availability of health care - Mean time to reach health-care facility negatively affect perception of health-care availability |
| Wagstaff A 2019 | 111 countries | RMNC, NCDs and SPA | Coverage and financial protection | Assessment of UHC | - UHC index varies (low in low-income countries and high in high-income countries) - Financial protection varied among countries |
| Wagstaff A 2015 | Latin America countries | RMNC and NCDs | Coverage | Assessing progress towards UHC | - No country has achieved UHC score |
| Zhang C et al 2019 | Ghana | RMNC, NCDs and CDs | Coverage and equity | Investigates the progress of UHC and its prediction up to 2030 | - Improvement in SC and FRP - Inequality will continue by 2030 |
| Ranjan A et al 2019 | India | CDs | Coverage | Measure current situation of UHC | - Unmet health care needs and lack of continuity of care |
| Fitzpatrick C et al 2018 | 123 countries | NTDs | Coverage and equity | Develop neglected tropical diseases (NTD) service coverage index | - NTD index can be used to measure equity in progress towards UHC |
| Eckhardt M et al 2018 | Ecuador | Emergency care | Financial protection | Healthcare-seeking behavior, determinants and health expenditure | - Provision of free health services may not sufficient to reach UHC |
| Suzana M et al 2018 | 39 small island developing states | SCA | Coverage | Empirical analysis of the potential impact of importing health services on UHC | - Importing health services helps to achieve UHC |
| Aantjes C et al 2016 | Zambia | Not specific | Non specific | Assesses successive Zambian governments’ efforts to achieve UHC | - Restructuring policies emphasised on social determinants of health and health equity - Policy and practice need to be extended to include ministries which focus on economic development |

CDs: communicable diseases; NCDs: non-communicable diseases; NTDs: neglected tropical diseases; RMNC: reproductive, maternal, neonatal and child health; SC: service coverage; SCA: Service Capacity and Access; UHC: Universal Health Coverage
